# HIV viral suppression and longevity among a cohort of children initiating antiretroviral therapy in Eastern Cape, South Africa

**DOI:** 10.1002/jia2.25168

**Published:** 2018-08-10

**Authors:** Chloe A Teasdale, Nonzwakazi Sogaula, Katharine A Yuengling, Chunhui Wang, Anthony Mutiti, Stephen Arpadi, Mahlubandile Nxele, Lungile Pepeta, Mary Mogashoa, Emilia D Rivadeneira, Elaine J Abrams

**Affiliations:** ^1^ ICAP at Columbia Mailman School of Public Health Columbia University New York NY USA; ^2^ Department of Epidemiology Mailman School of Public Health Columbia University New York NY USA; ^3^ Port Elizabeth Hospital Complex Port Elizabeth South Africa; ^4^ Faculty of Health Sciences Nelson Mandela University Port Elizabeth South Africa; ^5^ US Centers for Disease Control and Prevention Pretoria South Africa; ^6^ US Centers for Disease Control and Prevention Atlanta GA USA; ^7^ College of Physicians & Surgeons Columbia University New York NY USA

**Keywords:** viral load, viral load suppression, paediatric HIV, antiretroviral therapy, viral load rebound, late ART initiation

## Abstract

**Introduction:**

There are limited data on viral suppression (VS) in children with HIV receiving antiretroviral therapy (ART) in routine care in low‐resource settings. We examined VS in a cohort of children initiating ART in routine HIV care in Eastern Cape Province, South Africa.

**Methods:**

The Pediatric Enhanced Surveillance Study enrolled HIV‐infected ART eligibility children zero to twelve years at five health facilities from 2012 to 2014. All children received routine HIV care and treatment services and attended quarterly study visits for up to 24 months. Time to VS among those starting treatment was measured from ART start date to first viral load (VL) result <1000 and VL <50 copies/mL using competing risk estimators (death as competing risk). Multivariable sub‐distributional hazards models examined characteristics associated with VS and VL rebound following suppression among those with a VL >30 days after the VS date.

**Results:**

Of 397 children enrolled, 349 (87.9%) started ART: 118 (33.8%) children age <12 months, 122 (35.0%) one to five years and 109 (31.2%) six to twelve years. At study enrolment, median weight‐for‐age z‐score (WAZ) was −1.7 (interquartile range (IQR):−3.1 to −0.4) and median log VL was 5.6 (IQR: 5.0 to 6.2). Cumulative incidence of VS <1000 copies/mL at six, twelve and twenty‐four months was 57.6% (95% CI 52.1 to 62.7), 78.7% (95% CI 73.7 to 82.9) and 84.0% (95% CI 78.9 to 87.9); for VS <50 copies/mL: 40.3% (95% CI 35.0 to 45.5), 63.9% (95% CI 58.2 to 69.0) and 72.9% (95% CI 66.9 to 78.0). At 12 months only 46.6% (95% CI 36.6 to 56.0) of children <12 months had achieved VS <50 copies/mL compared to 76.9% (95% CI 67.9 to 83.7) of children six to twelve years (*p *<* *0.001). In multivariable models, children with VL >1 million copies/mL at ART initiation were half as likely to achieve VS <50 copies/mL (adjusted sub‐distributional hazards 0.50; 95% CI 0.36 to 0.71). Among children achieving VS <50 copies/mL, 37 (19.7%) had VL 50 to 1000 copies/mL and 31 (16.5%) had a VL >1000 copies/mL. Children <12 months had twofold increased risk of VL rebound to VL >1000 copies/mL (adjusted relative risk 2.03, 95% CI: 1.10 to 3.74) compared with six to twelve year olds.

**Conclusions:**

We found suboptimal VS among South African children initiating treatment and high proportions experiencing VL rebound, particularly among younger children. Greater efforts are needed to ensure that all children achieve optimal outcomes.

## 1 Introduction

Access to early testing for HIV‐exposed infants and to antiretroviral therapy (ART) for children living with HIV has expanded globally; however, significant gaps exist across the testing to treatment cascade 1, 2. In 2015, less than half of children born to HIV‐infected mothers received diagnostic testing within two months of birth and treatment coverage remained well below global targets, with only 43% of 2.1 million children living with HIV globally receiving ART in 2016 3. Although overall progress has not met expectations, some countries have made significant gains towards achieving the UNAIDS 90‐90‐90 targets for testing, treatment and virological suppression 4. In South Africa, almost all HIV‐exposed children reportedly received HIV testing within the first two months of life (>95%) and 74% of children in need of ART were on treatment in 2015 2. There is less information on rates of viral suppression (VS) for children initiated on ART, particularly in resource‐limited settings (RLS). In a meta‐analysis of studies of children from low‐ and middle‐income countries, among children started on ART after 2009, only 72.7% (95% CI 62.6 to 82.8) achieved VS within 12 months 5.

One of the challenges to achieving optimal treatment outcomes for children in RLS is late initiation of ART 6, 7, 8. Children with advanced disease at ART initiation are less likely to achieve VS and have higher mortality 8, 9, 10. While countries have expanded early infant diagnosis (EID) services, many children continue to be missed and are enrolling late in care 1, 10, 11, 12, 13, 14. In addition to late enrolment, differences have also been observed in rates of VS by age at ART initiation. Several studies have shown that time to VS is often prolonged in infants and younger children and that infants are less likely to fully suppress 9, 15, 16, 17. High pretreatment viral loads (VL) and suboptimal antiretroviral formulations and regimens for this age group likely contribute to poor outcomes 17, 18, 19.

The *Enhanced Surveillance and two Year Outcomes of Children Enrolled on ART in Public Health Facilities in the Eastern Cape Province, South Africa* (PESS) study followed a cohort of ART‐eligible children enrolled in routine HIV care and treatment services for up to 24 months at five public health facilities. We report rates of VS among children starting treatment and longevity of VS, as well as enrolment characteristics associated with suppression and VL rebound.

## 2 Methods

PESS was an observational cohort study conducted from 2012 through 2015 at five health facilities in Eastern Cape province, South Africa (facilities were a convenience sample selected to reflect service at tertiary hospitals and community clinics). It enrolled HIV‐infected, ART‐naïve children from birth through 12 years of age at the time of ART eligibility. All children identified as ART eligible by healthcare providers were offered study enrolment and followed up for 12 to 24 months. Caregivers provided consent (assent taken from children ≥8 years). Ethical and administrative reviews were received from the Columbia University Medical Center institutional review board, University of Cape Town Human Research Ethics Committee, US Centers for Disease Control and Prevention (CDC) Center for Global Health Associate Director for Science Office, East London Hospital Complex Research Ethics Committee, Walter Sisulu University Health Research Ethics Committee and Eastern Cape Department of Health.

All children enrolled in PESS received routine HIV care at participating facilities including ART, opportunistic infection prophylaxis and treatment, and routine laboratory monitoring (study did not provide medical care). Data on the child's health status, medical treatment and routine laboratory results were extracted from paper‐based medical records. South African national paediatric ART eligibility and laboratory‐monitoring guidelines changed during the study. Per 2010 national guidelines, all children <12 months were eligible for ART; children one to five years were eligible if they had World Health Organization (WHO) clinical Stage 3 or 4, CD4+ cell count (CD4+) <25% or absolute CD4+ count <750 cells/mm^3^; children >5 years were subject to adult guidelines of CD4 < 350 cells/mm^3^ or WHO Stage 3 or 4. In 2013, guidelines changed to ART for all children <5 years and five to fifteen year olds with WHO Stage 3 or 4 or CD4+ ≤350 cells/mm^3^ 20, 21. For laboratory monitoring, 2010 guidelines called for CD4+ and VL measurement at ART initiation, six and twelve months on treatment and then annually. The 2013 guidelines called for CD4+ at initiation, 12 months and then annually with VL measured every six months following ART initiation for children <5 years and annually starting at 12 months for children five to fifteen years. The first‐line ART regimen for children <3 years was abacavir (ABC), lamivudine (3TC) and lopinavir/ritonavir (LPV/r) and for children three years and older, ABC, 3TC and efavirenz (EFV) 20, 21. The study relied on routinely conducted laboratory tests, including CD4+ (MPL 1, Beckman Coulter) and VL (Cobas© 6800/8800, Roche); however, children had a final study‐related VL at their last study visit (which ranged from 12 to 24 months). All laboratory testing was performed by the National Health laboratories at Livingstone and Dora Nginza Hospitals in Port Elizabeth and at Frere and Cecilia Makiwane Hospitals in East London.

Children enrolled in PESS attended quarterly study visits, co‐scheduled with monthly routine care visits, which included caregiver questionnaires regarding the child's health status, family/life events and reported medication adherence. Data collected for the study were added to children's medical records. The study actively tracked enrolled children who missed a study visit through phone calls to caregivers and home visits to find children who had not returned for a scheduled visit. Information on deaths came from medical records, death certificates and verbal autopsies.

We examined rates of VS for children who initiated ART during study follow‐up. Enrolment characteristics included hospitalization at enrolment, history of hospitalizations (>30 days prior to enrolment date), tuberculosis (TB) at enrolment (as documented in the clinical chart up to 90 days prior to enrolment), ever having had TB (diagnosis in chart >90 days before enrolment or reported by caregiver), CD4+ and VL at enrolment (up to one year prior or one month after enrolment) and maternal age and history of prevention of mother‐to‐child transmission (PMTCT) interventions. We examined two outcomes, VS <1000 copies/mL and VS <50 copies/mL. Time to VS was measured from ART initiation date to date of the child's first suppressed VL at the two thresholds. We included all VL taken after ART initiation using time windows to examine proportions of children with VL measures at six months (2 to <9 months from ART start date), twelve months (9 to <18 months) and twenty‐four months (18 to <25 months); when more than one measurement was available, the closest to the six‐, twelve‐ and twenty‐four‐month time point was used. We used competing risk estimators to assess the cumulative incidence of VS, treating death as a competing risk. Multivariable sub‐distributional hazards models were used to identify factors associated with VS.

To assess longevity of VS, we examined the proportion of children who achieved VS and had at least one subsequent VL measure >30 days after the date of VS. VL rebound was defined as a VL measure >1000 copies/mL and for children with VS <50 copies/mL, we also looked at subsequent VL measures 50 to 1000 copies/mL. We examined enrolment characteristics associated with VL rebound among children who achieved VS <1000 copies/mL and <50 copies/mL using multivariable modified Poisson regression models to estimate relative risk. All multivariable models were adjusted for enrolment characteristics selected *a priori* (age, gender, maternal PMTCT, primary caregiver, maternal age, weight‐for‐age z‐score (WAZ), VL, CD4+ and for intra‐site clustering across the five facilities.

## 3 Results

Among 446 HIV‐infected children aged <12 years identified by healthcare providers as eligible for ART, 401 caregivers provided consent and 397 ART‐eligible children enrolled (four children were ineligible based on prior ART). Of 397 children enrolled, 35 (8.8%) died, 49 (12.3%) withdrew (in almost all cases because the child moved to a different health facility for care) and 6 (1.5%) children were lost to follow‐up. Overall, 349 (87.9%) started ART during study follow‐up: 118 (33.8%) children <12 months, 122 (35.0%) children one to five years and 109 (31.2%) six to twelve years. Among 48 children who did not start ART; twenty‐five (52.1%) died, two (4.2%) were loss to follow‐up (LTF) and twenty‐one (43.8%) transferred to another health facility prior to ART initiation. The focus of this analysis is on children who started ART. Median time on study (from enrolment) for children initiating ART was 23 months (interquartile range (IQR) 15 to 24) and median time from enrolment to ART initiation was 12 days (IQR 0 to 27). Median age at HIV diagnosis among children in this analysis, as documented in the medical record or reported by the caregiver, was 18 months (IQR 3 to 60); among children who were six to twelve years at study enrolment, 63.3% were not diagnosed until >5 years. At study enrolment, 38.1% of children were hospitalized; among those, 41 (30.8%) and 17 (12.8%) had a WHO Stage 3 or 4 condition respectively. Eighty‐three children (23.8%) had TB at the time of enrolment and 138 (39.5%) had ever had TB. Most children (70.2%) had mothers as their primary caregivers. While almost all children (91.4%) lived in a home with electricity, only 58.5% had a tap inside the home (Table 1). At study enrolment, median WAZ was −1.7 (IQR −3.1 to −0.4) and median log VL was 5.6 (IQR 5.0 to 6.2); log VL was significantly higher among children <12 months compared to older children (*p* < 0.0001) (Table 1). Almost all children started a first‐line regimen that included ABC, 3TC and LPV/r for children <3 years or EFV for older children; four children had missing ART regimen data.

**Table 1:** Characteristics at study enrollment among South African HIV‐infected children who started ART, PESS study 2012 to 2015 (N = 349)

	Age at enrollment								
	All		0 to 11 months		1 to 5 years		6 to 12 years		
	N	%	N	%	N	%	N	%	*p* value
	349	100.0	118	33.8	122	35.0	109	31.2	
Age at HIV diagnosis, median months (IQR)	18 (3 to 60)		2 (2 to 4)		19 (13 to 38)		88 (53 to 112)		<0.0001
0 to 5 months	114	32.7	99	83.9	11	9.0	4	3.7	<0.0001
6 to 11 months	31	8.9	19	16.1	10	8.2	2	1.8	
12 to 23 months	55	15.8			51	41.8	4	3.7	
2 to 3 years	38	10.9			26	21.3	12	11.0	
4 to 5 years	41	11.7			23	18.9	18	16.5	
6 to 11 years	70	20.1			1	0.8	69	63.3	
Female	169	48.4	53	44.9	61	50.0	55	50.5	0.64
Child hospitalized at enrollment	133	38.1	50	42.4	49	40.2	34	31.2	0.19
Child hospitalized ever	205	58.7	66	55.9	82	67.2	57	52.3	0.05
Child TB at enrollment	83	23.8	13	11.0	36	29.5	34	31.2	<0.01
Child TB ever	138	39.5	19	16.1	62	50.8	57	52.3	<0.0001
Mother PMTCT, reported or recorded									
Single dose NVP	23	6.6	5	4.2	12	9.8	6	5.5	<0.0001
AZT/NVP	52	14.9	30	25.4	20	16.4	2	1.8	
ART	64	18.3	41	34.7	18	14.8	5	4.6	
Other	3	0.9	3	2.5	0	0.0	0	0.0	
No PMTCT	158	45.3	36	30.5	49	40.2	73	67.0	
No information	49	14.0	3	2.5	23	18.9	23	21.1	
Mother alive at enrollment	298	85.4	116	98.3	106	86.9	76	69.7	<0.0001
Mother ≤25 years at enrollment	88	25.2	52	44.1	35	28.7	1	0.9	<0.0001
Primary caregiver									
Mother	245	70.2	102	86.4	78	63.9	65	59.6	0.0001
Grandmother	52	14.9	10	8.5	21	17.2	21	19.3	
Other	52	14.9	6	5.1	23	18.9	23	21.1	
Inside tap in home	222	63.6	69	58.5	73	59.8	80	73.4	0.037
Electricity in home	319	91.4	107	90.7	110	90.2	102	93.6	0.61
Enrollment WAZ, median (IQR)	−1.7 (−3.1 to −0.4)		−1.4 (−2.9 to 0.1)		−2.1 (−3.6 to −0.5)		−1.7 (−3.0 to −0.7)		0.09
<−2	145	45.3	38	37.6	62	53.9	45	43.3	0.05
−2 to −1	56	17.5	19	18.8	13	11.3	24	23.1	
>−1	119	37.2	44	43.6	40	34.8	35	33.7	
Missing	29	8.3	17	14.4	7	5.7	5	4.6	0.01
Enrollment VL copies/mL, median (IQR)	426,550 (92,654 to 1738,371)		1341,451 (450,421 to 4983,120)		415,979 (115,317 to 1580,291)		115,769 (42,890 to 375,770)		<0.0001
>1 million	108	36.4	63	60.6	36	34.6	9	10.1	<0.0001
100,000 to 1 million	110	37.0	28	26.9	45	43.3	37	41.6	
10,000 to 99,999	63	21.2	9	8.7	19	18.3	35	39.3	
<10,000	16	5.4	4	3.8	4	3.8	8	9.0	
Missing	52	14.9	14	11.9	18	14.8	20	18.3	0.39
Enrollment VL (log), median (IQR)	5.6 (5.0 to 6.2)		6.1 (5.7 to 6.7)		5.6 (5.1 to 6.2)		5.1 (4.6 to 5.6)		<0.0001
>6.0	108	36.4	63	60.6	36	34.6	9	10.1	<0.0001
5.0 to 6.0	110	37.0	28	26.9	45	43.3	37	41.6	
4.0 to 4.9	63	21.2	9	8.7	19	18.3	35	39.3	
<4.0	16	5.4	4	3.8	4	3.8	8	9.0	
Missing	52	14.9	14	11.9	18	14.8	20	18.3	0.39
Enrollment CD4 count, cells/mm^3^ median (IQR)	565 (308 to 1138)		1284 (578 to 1746)		645 (34 to 1086)		338 (204 to 528)		<0.0001
>1000	92	30.1	56	57.7	29	27.9	7	6.7	<0.0001
500 to 1000	77	25.2	21	21.6	36	34.6	20	19.0	
350 to 500	46	15.0	10	10.3	13	12.5	23	21.9	
200 to 350	45	14.7	2	2.1	14	13.5	29	27.6	
<200	46	15.0	8	8.2	12	11.5	26	24.8	
Missing	43	12.3	21	17.8	18	14.8	4	3.7	0.003
Enrollment CD4%, median (IQR)	17.4 (11.2 to 25.7)		21.1 (13.8 to 30.3)		16.3 (11.2 to 22.8)		15.3 (7.7 to 23.2)		<0.0001
>40%	15	5.0	11	11.2	4	3.9	0	0.0	0.0008
25% to 40%	69	22.8	30	30.6	18	17.6	21	20.4	
15% to 25%	95	31.4	27	27.6	37	36.3	31	30.1	
<15%	124	40.9	30	30.6	43	42.2	51	49.5	
Missing	46	13.2	20	16.9	20	16.4	6	5.5	0.02
First regimen (N = 349)									
ABC+3TC+EFV	138	40.0	0	0.0	37	30.8	101	93.5	<0.0001
ABC+3TC+LPV/r	205	59.4	116	99.1	83	69.2	6	5.6	
ABC+3TC+NVP	1	0.3	0	0.0	0	0.0	1	0.9	
AZT+3TC+LPV/r	1	0.3	1	0.9	0	0.0	0	0.0	
Missing	4	1.1	1	0.8	2	1.6	1	0.9	0.82

PESS, Pediatric Enhanced Surveillance Study; IQR, interquartile range (IQR); TB, tuberculosis; NVP, nevirapine; AZT, azidothymidine; PMTCT, prevention of mother‐to‐child transmission (of HIV); WAZ, weight‐for‐age z score; VL, viral load; ABC, abacavir; 3TC, lamivudine; LPV/r, lopinavir/ritonavir; EFV, efavirenz.

Among the 118 infants (<12 months) who started ART, there were 37 (31.4%) zero to three months, 45 (38.1%) three to five months and 36 (30.5%) six to eleven months of age (Table 2). Almost half (42.4%) of the children <12 months were hospitalized at enrolment and 11.0% had TB at enrolment. A third of all mothers of younger children (34.8%) had received ART for PMTCT and 30.5% of mothers received no ARVs for PMTCT (2.5% had missing PMTCT information) (Table 2). Among the younger children, median WAZ was −1.4 (IQR −2.9 to 0.1) and was lowest, −1.9 (IQR −3.6 to 0.0), among children three to five months (*p *=* *0.03). The median log VL was 6.1 (IQR 5.7 to 6.7). All children <12 months started first‐line treatment with LPV/r (one child missing regimen data) (Table 2).

**Table 2:** Characteristics at study enrollment among South African HIV‐infected children <12 months who started ART, PESS study 2012 to 2015 (N = 118)

	Age at enrollment								
	All		<3 months		3 to 5 months		6 to 11 months		
	N	%	N	%	N	%	N	%	*p* value
	118	100.0	37	31.4	45	38.1	36	30.5	
Age at HIV diagnosis, median months (IQR)	2.0 (1.6 to 4.0)		1.6 (1.0 to 2.0)		2.8 (2.0 to 3.0)		6.0 (3.3 to 9.5)		<0.0001
0 to 5 months	99	83.9	37	100.0	45	100.0	17	47.2	<0.0001
6 to 11 months	19	16.1	0	0.0	0	0.0	19	52.8	
Female	53	44.9	17	46.0	22	48.9	14	38.9	0.66
Child hospitalized at enrollment	50	42.4	15	40.5	19	42.2	16	44.4	0.94
Child hospitalized ever	66	55.9	18	48.6	28	62.2	20	55.6	0.47
Child TB at enrollment	13	11.0	0	0.0	4	8.9	9	25.0	0.003
Child TB ever	19	16.1	0	0.0	8	17.8	11	30.6	<0.01
Mother PMTCT, reported or recorded									
Single dose NVP	5	4.2	1	2.7	3	6.7	1	2.8	0.08
AZT/NVP	30	25.4	4	10.8	15	33.3	11	30.6	
ART	41	34.7	17	45.9	17	37.8	7	19.4	
Other	3	2.5	0	0.0	1	2.2	2	5.6	
No PMTCT	36	30.5	14	37.8	9	20.0	13	36.1	
No information	3	2.5	1	2.7	0	0.0	2	5.7	
Mother alive at enrollment	116	98.3	36	97.3	44	97.8	36	100.0	0.63
Mother ≤25 years at enrollment	52	44.1	12	32.4	24	53.3	16	44.4	0.34
Primary caregiver									
Mother	102	86.4	32	86.5	41	91.1	29	80.6	0.36
Grandmother	10	8.5	4	10.8	3	6.7	3	8.3	
Other	6	5.1	1	2.7	1	2.2	4	11.1	
Inside tap in home	69	58.5	25	67.6	26	57.8	18	50.0	0.31
Electricity in home	107	90.7	35	94.6	40	88.9	32	88.9	0.61
Enrollment WAZ, median (IQR)	−1.4	(−2.9 to 0.1)	−0.5	(−1.8 to 0.3)	−1.9	(−3.6 to 0.0)	−1.6	(−2.9 to −0.2)	0.03
<−2	38	37.6	6	18.8	19	48.7	13	43.3	0.10
−2 to −1	19	18.8	8	25.0	5	12.8	6	20.0	
>−1	44	43.6	18	56.3	15	38.5	11	36.7	
Missing	17	14.4	5	13.5	6	13.3	6	16.7	0.90
Enrollment VL copies/mL, median (IQR)	1341,451 (450,421 to 4983,120)		1239,375 (324,340 to 4745,448)		1979,259 (537,330 to 5172,660)		1011,080 (433,902 to 3516,790)		0.71
>1 million	63	60.6	20	62.5	25	64.1	18	54.5	0.27
100,000 to 1 million	28	26.9	6	18.8	9	23.1	13	39.4	
10,000 to 99,999	9	8.7	3	9.4	4	10.3	2	6.1	
<10,000	4	3.8	3	9.4	1	2.6	0	0.0	
Missing	14	11.9	5	13.5	6	13.3	3	8.3	0.73
Enrollment VL (log), median (IQR)	6.1 (5.7 to 6.7)		6.1 (5.5 to 6.7)		6.3 (5.7 to 6.7)		6.0 (5.6 to 6.5)		0.71
>6.0	63	60.6	20	62.5	25	64.1	18	54.5	0.27
5.0 to 6.0	28	26.9	6	18.8	9	23.1	13	39.4	
4.0 to 4.9	9	8.7	3	9.4	4	10.3	2	6.1	
<4.0	4	3.8	3	9.4	1	2.6	0	0.0	
Missing	14	11.9	5	13.5	6	13.3	3	8.3	0.73
Enrollment CD4 count cells/mm^3^, median (IQR)	1284 (578 to 1746)		1509 (473 to 2243)		1396 (518 to 1734)		1071 (730 to 1622)		0.07
>1000	56	57.7	16	55.2	23	62.2	17	54.8	0.07
500 to 1000	21	21.6	4	13.8	5	13.5	12	38.7	
350 to 500	10	10.3	6	20.7	3	8.1	1	3.2	
200 to 350	2	2.1	1	3.4	1	2.7	0	0.0	
<200	8	8.2	2	6.9	5	13.5	1	3.2	
Missing	21	17.8	8	21.6	8	17.8	5	13.9	0.69
Enrollment CD4%, median (IQR)	21.1 (13.8 to 30.3)		20.0 (15.2 to 37.8)		22.8 (13.8 to 30.5)		19.4 (12.6 to 27.7)		0.63
>40%	11	11.2	5	17.2	3	7.9	3	9.7	0.58
25% to 40%	30	30.6	7	24.1	15	39.5	8	25.8	
15% to 25%	27	27.6	10	34.5	8	21.1	9	29.0	
<15%	30	30.6	7	24.1	12	31.6	11	35.5	
Missing	20	16.9	8	21.6	7	15.6	5	13.9	0.65
First regimen									
ABC+3TC+LPV/r	116	99.1	36	100.0	45	100.0	35	97.2	0.32
AZT+3TC+LPV/r	1	0.9	0	0.0	0	0.0	1	2.8	
Missing	1	0.8	1	2.7	0	0.0	0	0.0	0.33

PESS, Pediatric Enhanced Surveillance Study; IQR, interquartile range (IQR); TB, tuberculosis; NVP, nevirapine; AZT, azidothymidine; PMTCT, prevention of mother‐to‐child transmission (of HIV); WAZ, weight‐for‐age z score; VL, viral load; ABC, abacavir; 3TC, lamivudine; LPV/r, lopinavir/ritonavir; ART, antiretroviral therapy.

Most children who started ART had VL measures taken at six and twelve months (73.9% and 80.2% respectively) and 35.0% had a VL at 24 months (Table 3). The overall cumulative incidence of VS <1000 copies/mL at six, twelve and twenty‐four months was 57.6% (95% CI 52.1 to 62.7), 78.7% (95% CI 73.7 to 82.9) and 84.0% (95% CI 78.9 to 87.9); for VS <50 copies/mL the cumulative incidence was 40.3% (95% CI 35.0 to 45.5), 63.9% (95% CI 58.2 to 69.0) and 72.9% (95% CI 66.9 to 78.0) (Table 4). Children <12 months had lower rates of VS; at 12 months only 68.0% (95% CI 58.2 to 75.9) had achieved VS <1000 copies/mL and less than half, 46.6% (95% CI 36.6 to 56.0) had reached VS <50 copies/mL, compared to children six to twelve years, among whom 83.9% (95% CI 75.6 to 89.6) had achieved VS <1000 copies/mL and 76.9% (95% CI 67.9 to 83.7) had VS <50 copies by 12 months (*p* = 0.001; *p* < 0.001; Table 4). Among infants in the study, the age category three to five months had the lowest rates of VS <50 copies/mL; only 41.2% (95% CI 25.5 to 56.2) of these infants achieved VS <50 copies/mL compared to 55.2% (95% CI 36.5 to 70.5) of children <3 months and 76.9% (95% CI 67.9 to 83.7) of children six to twelve years (*p *<* *0.001). Children starting a first‐line regimen containing LPV/r (those <3 years) were also less likely to achieve VS <50 copies compared to those starting an EFV‐based regimen (>3 years) (*p *<* *0.001) (Table 4).

**Table 3:** VL testing data by age group among South African HIV‐infected children who started ART, PESS study 2012 to 2015 (N = 349)

Age group	6 month VL	12 month VL	24 month VL
All (N = 349)	258 (73.9)	280 (80.2)	122 (35.0%)
0 to 11 months (N = 118)	77 (65.3)	88 (74.6)	42 (36.0)
1 to 5 years (N = 122)	94 (77.0)	100 (82.0)	40 (32.8)
6 to 12 (N = 109)	87 (79.8)	92 (84.4)	40 (36.7)

ART, antiretroviral therapy; VL, viral load.

**Table 4:** Cumulative incidence of VS among South African children zero to twelve years of age starting ART, PESS study 2012 to 2015 (N = 349)

	6 months		12 months		24 months		*p*‐value
	Cum. Inc.	95% CI	Cum. Inc.	95% CI	Cum. Inc.	95% CI	
VS <1000 copies/mL							
Overall VS<1000 copies/mL	57.6	52.1 to 62.7	78.7	73.7 to 82.9	84.0	78.9 to 87.9	
Age at enrolment							
<3 months	46.9	29.7 to 62.4	68.1	49.5 to 81.1	78.1	56.9 to 89.7	0.001
3 to 5 months	31.8	18.8 to 45.6	67.0	50.3 to 79.2	73.3	56.3 to 84.6	
6 to <12 months	50.7	32.9 to 66.1	69.9	50.7 to 82.8	75.9	54.4 to 88.3	
<12 months overall	42.2	32.9 to 51.1	68.0	58.2 to 75.9	76.3	65.7 to 84.1	
1 to 5 years	59.9	49.7 to 68.6	85.0	75.6 to 91.0	89.4	79.2 to 94.8	
6 to 12 years	69.9	60.8 to 77.4	83.9	75.6 to 89.6	87.4	79.3 to 92.5	
First‐line regimen							
ABC+3TC+EFV (N = 138)	70.6	62.1 to 77.5	85.1	77.6 to 90.3	88.0	80.7 to 92.6	0.03
ABC+3TC+LPV/r (N = 205)	48.5	41.3 to 55.3	74.5	67.5 to 80.2	81.9	74.2 to 87.4	
VS <50 copies/mL							
Overall VS < 50 copies/mL	40.3	35.0 to 45.5	63.9	58.2 to 69.0	72.9	66.9 to 78.0	
Age at enrolment							
Age at enrolment							
<3 months	29.4	15.4 to 44.9	55.2	36.5 to 70.5	60.2	40.2 to 75.4	<0.001
3 to 5 months	13.8	5.6 to 25.6	41.2	25.5 to 56.2	57.2	37.9 to 72.5	
6 to <12 months	17.9	7.3 to 32.4	45.0	26.9 to 61.5	61.4	38.4 to 78.0	
<12 months overall	19.8	13.0 to 27.7	46.6	36.6 to 56.0	60.8	48.6 to 71.0	
1 to 5 years	40.4	30.9 to 49.7	67.5	56.8 to 76.1	74.6	63.3 to 82.9	
6 to 12 years	59.5	50.0 to 67.7	76.9	67.9 to 83.7	83.0	74.1 to 89.1	
First‐line regimen							
ABC+3TC+EFV (N = 138)	58.4	49.5 to 66.2	78.2	70.0 to 84.5	84.3	76.3 to 89.7	<0.001
ABC+3TC+LPV/r ((N = 205)	28.1	22.0 to 34.5	53.7	46.0 to 60.8	65.1	56.2 to 72.7	

PESS, Pediatric Enhanced Surveillance Study; Cum. Inc., Cumulative Incidence; 95% CI, 95% confidence interval; ART, antiretroviral therapy; VS, viral suppression; ABC, abacavir; 3TC, lamivudine; LPV/r, lopinavir/ritonavir; EFV, efavirenz.

In univariable models, age <12 months was significantly associated with VS <1000 copies/mL and VS <50 copies/mL; however, after adjustment for other covariates, including VL at study enrolment, the effect was no longer significant (Table 5). In multivariable models, lower VL at enrolment was a strong predictor of VS. Children with VL >1 million copies/mL at ART initiation were 43% less likely to achieve VS <1000 copies/mL compared to children with <10,000 copies/mL (adjusted sub‐distributional hazards ratio (aSHR) 0.55; 95% CI 0.44 to 0.68) and half as likely to achieve VS <50 copies/mL (aSHR 0.52; 95% CI 0.36 to 0.74) (Table 5). Younger maternal age (≤25 years) was also associated with a lower incidence of VS <50 copies/mL in children (aSHR 0.58; 95% CI 0.49 to 0.68) (Table 5).

**Table 5:** Enrollment characteristics associated with VS among South African children zero to twelve years starting ART, PESS study 2012 to 2015 (N = 349)

	VS <1000 copies/mL						Viral suppression <50 copies/mL					
	Univariable			Multivariable			Univariable			Multivariable		
	SHR	95% CI	*p*‐value	aSHR	95% CI	*p*‐value	SHR	95% CI	*p*‐value	aSHR	95% CI	*p*‐value
Age at enrollment												
<12 months	1	ref		1	ref		1	ref		1	ref	
1 to 5 years	1.50	1.19 to 1.88	<0.001	1.04	0.91 to 1.20	0.56	1.70	1.25 to 2.30	0.001	1.23	0.84 to 1.79	0.28
6 to 12 years	1.67	1.25 to 2.22	<0.001	1.03	0.68 to 1.53	0.90	2.29	1.64 to 3.18	<0.001	1.18	0.79 to 1.77	0.42
Female	1.07	0.82 to 1.40	0.60	1.10	0.94 to 1.27	0.23	1.19	0.71 to 2.00	0.52	1.21	0.88 to 1.67	0.24
Mother PMTCT												
ART	1	ref		1	ref		1	ref		1	ref	
Single dose dNVP	1.32	0.60 to 2.88	0.49	1.24	0.65 to 2.35	0.52	1.24	0.83 to 1.86	0.30	1.24	0.61 to 2.54	0.55
AZT/NVP	1.44	1.14 to 1.80	0.002	1.47	1.22 to 1.78	<0.001	1.50	0.72 to 3.12	0.28	1.52	0.80 to 2.88	0.20
Other	1.28	0.87 to 1.88	0.21	1.50	1.04 to 2.16	0.03	1.04	0.18 to 6.18	0.96	1.39	0.39 to 4.92	0.61
No PMTCT	2.03	1.29 to 3.17	0.002	1.88	1.06 to 3.34	0.03	2.03	1.47 to 2.79	<0.001	1.96	1.09 to 3.54	0.02
Missing	3.08	1.97 to 4.81	<0.001	2.12	1.19 to 3.77	0.01	3.20	1.73 to 5.92	<0.001	2.88	1.02 to 8.11	0.05
Mother as primary caregiver	0.56	0.45 to 0.69	<0.001	0.70	0.49 to 0.99	0.05	0.62	0.39 to 1.01	0.06	0.94	0.61 to 1.46	0.79
Mother age ≤25 years	0.90	0.66 to 1.24	0.53	0.85	0.71 to 1.03	0.09	0.61	0.49 to 0.76	<0.001	0.58	0.49 to 0.68	<0.001
WAZ												
<−2	0.82	0.63 to 1.08	0.17	0.94	0.74 to 1.18	0.58	0.73	0.57 to 0.93	0.01	0.71	0.51 to 1.00	0.05
−2 to −1	0.86	0.57 to 1.30	0.48	0.96	0.63 to 1.44	0.83	0.93	0.73 to 1.19	0.59	0.91	0.72 to 1.15	0.42
>−1	1	ref		1	ref		1	ref		1	ref	
Missing	0.59	0.48 to 0.72	<0.001	0.65	0.54 to 0.79	<0.001	0.66	0.53 to 0.84	0.001	0.68	0.44 to 1.07	0.10
TB at enrollment	0.96	0.83 to 1.10	0.52	0.95	0.76 to 1.19	0.65	1.00	0.73 to 1.38	0.99	0.90	0.61 to 1.35	0.62
Enrollment VL copies/mL												
>1 million	0.40	0.31 to 0.51	<0.001	0.55	0.44 to 0.68	<0.001	0.32	0.21 to 0.51	<0.001	0.52	0.36 to 0.74	<0.001
100,000 to 1 million	0.60	0.45 to 0.80	<0.001	0.69	0.54 to 0.90	0.01	0.66	0.52 to 0.84	0.001	0.84	0.58 to 1.20	0.33
10,000 to 99,999	0.78	0.56 to 1.08	0.13	0.79	0.63 to 0.98	0.03	0.91	0.64 to 1.31	0.62	0.98	0.67 to 1.45	0.94
<10,000	1	ref		1	ref		1	ref		1	ref	
Missing	0.61	0.45 to 0.83	0.002	0.65	0.39 to 1.08	0.10	0.52	0.39 to 0.71	<0.001	0.54	0.31 to 0.96	0.03
CD4 count cells/mm^3^												
<200	0.92	0.69 to 1.23	0.57	0.89	0.69 to 1.15	0.37	1.14	0.70 to 1.86	0.60	1.09	0.71 to 1.67	0.004
200 to 350	1.20	0.83 to 1.73	0.34	0.86	0.54 to 1.36	0.51	1.37	1.00 to 1.90	0.05	0.88	0.57 to 1.36	0.57
350 to 500	1.42	1.05 to 1.92	0.02	1.46	1.23 to 1.73	<0.001	1.70	1.06 to 2.73	0.03	1.54	1.12 to 2.12	0.01
500 to 1000	1.25	1.01 to 1.55	0.04	1.29	0.96 to 1.72	0.09	1.15	0.79 to 1.66	0.47	1.05	0.74 to 1.50	0.77
>1000	1	ref		1	ref		1	ref		1	ref	
Missing	1.32	1.15 to 1.52	<0.001	1.60	1.41 to 1.82	<0.001	1.18	0.73 to 1.93	0.50	1.61	1.17 to 2.22	0.70
CD4%^a^												
<15%	1.85	1.24 to 2.78	0.003				2.01	0.81 to 5.00	0.13			
15% to 25%	2.28	1.28 to 4.07	0.005				2.36	0.81 to 6.91	0.12			
25% to 40%	2.38	1.56 to 3.65	<0.001				2.49	1.00 to 6.21	0.05			
>40%	1	ref					1	ref				
Missing	2.52	1.73 to 3.69	<0.001				2.30	0.82 to 6.47	0.12			

SHR, sub‐distributional hazards ratio; 95% CI, 95% confidence interval; aSHR, adjusted sub‐distributional hazards ratio; VS, viral suppression; ART, antiretroviral therapy.

a CD4% not included in multivariable models.

**Figure 1:**
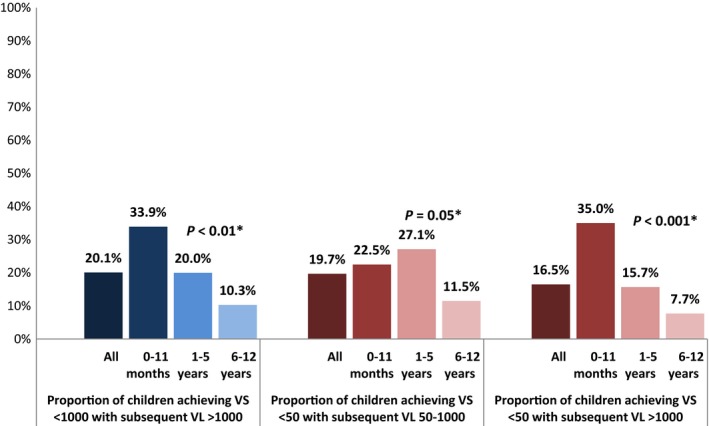
Proportion of South African children zero to twelve years starting antiretroviral therapy and achieving VS <1000 copies/mL (N = 234) and VS <50 copies/mL (N = 188) with subsequent viral load rebound, PESS study 2012 to 2015. *p‐value for chi‐square test comparing proportions of viral load rebound by age group. PESS, Pediatric Enhanced Surveillance Study; VS, viral suppression; VL, viral load.

Among 273 children achieving VS <1000 copies/mL, 234 (85.7%) had at least one VL measure after suppression and among those, 47 (20.1%) had VL rebound to >1000 copies/mL (Figure 1). The median time from VS to VL rebound was nine months (IQR 6 to 14). For the 229 children who suppressed to <50 copies/mL, 188 (82.1%) had a later VL, 37 (19.7%) had VL 50 to 1000 copies/mL and 31 (16.5%) had a VL >1000 copies/mL (nine children (4.8%) had both VL 50 to 1000 and >1000 copies/mL) (Figure 1). Median time from VS <50 copies to VL 50 to 1000 copies/mL was eight months (IQR 6 to 11) and to VL >1000 copies/mL was nine months (IQR 6 to 14). Among children <12 months who achieved VS <1000 copies/mL, almost 34% had VL rebound, compared to only 10% of children six to twelve years (*p *<* *0.01) (Figure 1). In multivariable models, children <12 months who achieved VS <50 copies/mL had twofold increased risk of VL rebound to VL >1000 copies/mL (adjusted relative risk 2.03, 95% CI: 1.10 to 3.74) compared to those six to twelve years (Table 6).

**Table 6:** Enrolment characteristics associated with VL rebound (VL > 1000 copies/mL) among South African children zero to twelve years starting ART with at least one VL after VS <1000 copies/mL (N = 234) and VS <50 copies/mL (N = 188), PESS study 2012 to 2015

	Children with VL rebound after VS <1000 copies/mL						Children with VL rebound after VS <50 copies/mL					
	Univariable			Multivariable			Univariable			Multivariable		
	RR	95% CI	*p*‐value	aRR	95% CI	*p*‐value	RR	95% CI	*p*‐value	aRR	95% CI	*p*‐value
Age at enrolment												
<12 months	3.26	2.19 to 4.84	<0.0001	1.61	0.81 to 3.22	0.17	4.53	2.16 to 9.51	<0.0001	2.03	1.10 to 3.74	0.02
1 to 5 years	1.93	1.18 to 3.13	0.008	1.58	0.80 to 3.11	0.19	2.05	0.99 to 4.23	0.05	1.43	0.70 to 2.93	0.32
6 to 12 years	1	ref		1	ref		1	ref		1	ref	
Female	1.11	0.50 to 2.47	0.79	1.12	0.64 to 1.95	0.69	0.84	0.26 to 2.70	0.77	0.68	0.21 to 2.14	0.51
Mother PMTCT												
Single‐dose NVP	1.00	0.66 to 1.50	1.00	1.48	0.59 to 3.69	0.41	0.91	0.57 to 1.45	0.69	1.60	0.66 to 3.91	0.30
AZT/NVP	1.06	0.31 to 3.61	0.92	1.05	0.39 to 2.84	0.92	1.30	0.41 to 4.18	0.66	1.18	0.54 to 2.58	0.67
ART	1	ref		1	ref		1	ref		1	ref	
No PMTCT	0.45	0.23 to 0.89	0.02	0.72	0.34 to 1.52	0.39	0.36	0.17 to 0.75	0.006	0.46	0.20 to 1.05	0.06
Missing	0.27	0.14 to 0.51	<0.0001	0.35	0.17 to 0.69	0.003	0.25	0.07 to 0.87	0.03	0.37	0.10 to 1.37	0.14
Mother as primary caregiver	1.30	0.99 to 1.72	0.06	0.98	0.50 to 1.93	0.96	1.70	0.95 to 3.04	0.07	0.99	0.48 to 2.06	0.99
Mother age >25 years	0.65	0.44 to 0.95	0.03	0.86	0.59 to 1.26	0.44	0.70	0.59 to 0.84	0.0001	0.88	0.74 to 1.03	0.12
WAZ^a^												
<−2	1.01	0.55 to 1.85	0.98	1.00	0.64 to 1.56	0.98						
−2 to −1	0.77	0.36 to 1.67	0.51	0.98	0.44 to 2.17	0.96						
>−1	1	ref		1	ref							
Missing	0.31	0.22 to 0.45	<0.0001	0.27	0.19 to 0.39	<0.0001						
TB at enrolment	0.68	0.53 to 0.87	0.002	0.99	0.71 to 1.38	0.96	0.81	0.56 to 1.17	0.26	1.21	0.68 to 2.14	0.51
Enrolment VL copies/mL												
>1 million	2.26	0.86 to 5.91	0.10	1.36	0.51 to 3.59	0.54	1.54	0.61 to 3.91	0.36	0.84	0.23 to 3.05	0.79
100,000 to 1 million	1.46	0.62 to 3.42	0.39	1.22	0.62 to 2.40	0.57	1.00	0.49 to 2.04	1.00	0.73	0.35 to 1.56	0.42
10,000 to 99,999	0.90	0.29 to 2.81	0.85	0.77	0.32 to 1.85	0.55	0.53	0.16 to 1.82	0.32	0.40	0.12 to 1.36	0.14
<10,000	1	ref		1	ref		1	ref		1	ref	
Missing	1.46	0.43 to 5.00	0.55	1.45	0.48 to 4.35	0.51	1.00	0.35 to 2.86	1.00	0.59	0.22 to 1.58	0.30
CD4 count cells/mm^3^												
>1000	1	ref		1	ref		1	ref		1	ref	
500 to 1000	0.37	0.23 to 0.59	<0.0001	0.43	0.30 to 0.62	<0.0001	0.17	0.07 to 0.39	<0.0001	0.25	0.17 to 0.37	<0.0001
350 to 500	0.20	0.09 to 0.49	0.0003	0.33	0.13 to 0.80	0.01	0.24	0.11 to 0.52	0.0003	0.44	0.14 to 1.40	0.17
200 to 350	0.56	0.44 to 0.72	<0.0001	0.88	0.75 to 1.03	0.11	0.49	0.30 to 0.79	0.003	1.03	0.46 to 2.31	0.94
<200	1.00	0.41 to 2.46	1.00	0.96	0.56 to 1.63	0.87	1.14	0.52 to 2.50	0.74	1.23	0.59 to 2.56	0.58
Missing	0.57	0.21 to 1.56	0.27	0.80	0.26 to 2.48	0.69	0.59	0.21 to 1.63	0.31	0.90	0.34 to 2.41	0.83
CD4%^b^												
>40%	1	ref					1	ref				
25% to 40%	0.43	0.15 to 1.26	0.12				0.62	0.09 to 4.03	0.61			
15% to 25%	0.42	0.15 to 1.24	0.12				0.36	0.09 to 1.42	0.14			
<15%	0.78	0.24 to 2.49	0.67				1.12	0.22 to 5.79	0.89			
Missing	0.49	0.29 to 0.81	0.006				0.75	0.28 to 2.03	0.57			

ART, antiretroviral therapy; VS, viral suppression; PESS, Pediatric Enhanced Surveillance Study; RR, relative risk; aRR, adjusted relative risk; NVP, nevirapine; AZT, azidothymidine; PMTCT, prevention of mother‐to‐child transmission (of HIV); WAZ, weight‐for‐age z‐score; VL, viral load.

a WAZ lacked sufficient variability for the VL rebound outcome for those with VS <50 copies/mL and was excluded from models.

b CD4% not included in multivariable models; #WAZ lacked sufficient variability for inclusion in models.

## 4 Discussion

In this cohort of 349 ART‐eligible children aged zero to twelve years receiving routine care and initiating ART at five health facilities in Eastern Cape, South Africa, we found that a majority of older children had late HIV diagnosis (>5 years of age) and, among all children, many had evidence of advanced disease at ART eligibility. Overall, more than three quarters of children achieved VS <1000 copies/mL by 12 months but fewer, 64%, achieved VS <50 copies/mL. We also found that children <12 months had lower rates of suppression compared with children six to twelve years. In multivariable models, age was not significantly associated with VS; however, higher VL predicted lower incidence of VS. Among children who achieved VS and had a subsequent VL, we found that 17% to 20% had a later VL measure >1000 copies/mL and, in multivariable models, age <12 months was associated with twice the risk of VL rebound.

This cohort illustrates that many children continue to be enrolled late in HIV care despite efforts to increase EID and HIV testing services. Among children six to twelve years at study enrolment, 63% were not diagnosed with HIV until they were five years or older. Furthermore, more than a third of children were hospitalized at enrolment, almost 60% had a history of hospitalization, close to 40% of children had TB or a history of TB and 45% had severely compromised growth (WAZ <−2). Similar characteristics indicating advanced disease at ART initiation have been found in South African paediatric cohorts, as well as those from other African countries 8, 9, 10, 12, 22. While this study included three hospital facilities where sicker children were more likely to seek care, our findings underscore the need to improve EID programmes and expand efforts to identify HIV‐infected children through routine opt‐out testing in all paediatric care settings, as well as through innovative approaches including family testing and non–facility‐based testing 14.

Almost 80% of children starting ART achieved VS <1000 copies/mL by 12 months, while the incidence of suppression <50 copies/mL was lower (64%). Overall VS in this cohort is somewhat lower than an earlier South African cohort in which 84% of children (median age 4.3 years) achieved VS <400 copies/mL by 12 months 9. Our results are similar to data from a pooled analysis of studies of children in RLS which found that 73% had suppressed to <1000 copies/mL by 12 months on ART and a Ugandan cohort which found 67% suppression (<400 copies/mL) at 12 months 5, 23. While most children in PESS achieved VS, those <12 months at ART initiation were less likely to; at 12 months, 84% of children six to twelve years had VS <1000 copies/mL compared to 68% of infants, and only 47% of infants had achieved VS <50 copies by 12 months compared to 77% of six to twelve year olds.

Although we did not find that age was a significant predictor of VS in multivariable models adjusted for clinical characteristics at ART initiation including VL, previous studies have found associations between younger age and lower incidence of suppression 16, 17, 23. Children with advanced disease at ART initiation take longer and are less likely to achieve VS; they are also at risk for having inadequate immune response to treatment 24. For the infants, the combination of advanced disease at ART initiation with immature immune system often leads to delayed VS particularly when adherence is inadequate and adequate drug levels are not well maintained 25, 26. Among infants starting ART at a median age of 5.9 months in pooled data from Southern Africa, only 28% and 56% achieved VS <400 copies/mL at six and twelve months 8. In our cohort, younger children had significantly higher VL at ART initiation compared to older children (61% of children <12 months had VL >1 million copies compared to 10% of six to twelve year olds) which has also been observed in other studies 17. In addition to higher VL, younger children may face greater adherence challenges as a result of medication syrups which can be difficult to administer and are highly unpalatable 19. While the incidence of VS <1000 copies/mL was similar for children one to five years and six to twelve years (85% and 84% at 12 months), for VS <50 copies/mL there were more pronounced differences with only 68% of one to five year olds achieving this outcome compared to 77% of older children. High rates of pretreatment HIV drug resistance, particularly nucleoside reverse transcriptase inhibitors and non‐nucleoside reverse transcriptase inhibitors mutations, have been found in HIV‐infected infants and children and can impact ART efficacy; however, it is unlikely to explain the differences observed by age in our study, as the youngest children, who had the lowest rates of suppression, received PI‐based regimens 27.

High VL at study enrolment was the only significant predictor of VS <1000 copies/mL and <50 copies/mL in multivariable models. Advanced disease, specifically high VL, has been associated with lower rates of VS in several other cohorts 16, 23. In addition to high VL, maternal age of 25 years or younger was also associated with lower incidence of VS <50 copies/mL. This finding is unique to our analysis and we were unable to find other studies that examined maternal age as a predictor of VS among children. Younger mothers were significantly more likely to have children <12 months of age who were, in turn, more likely to have high VL; however, even in multivariable models adjusted for child's age and VL, maternal age was still a significant predictor of VS. While lower VS for children with younger mothers has not been previously reported, existing data show that younger women are less likely to complete PMTCT and to be retained in care 28, 29. It is possible that the poor treatment outcomes among young mothers has an impact on their children's health; however, this question warrants further investigation. We also observed that children initiating treatment with a first regimen containing LPV/r were less likely to achieve VS; however, we could not examine this association in multivariable models as there was no variability in regimen by age (i.e. all children <3 years received a LPV/r regimen).

In our analysis of VL rebound after suppression, we found that 20% of children who achieved VS <1000 copies/mL had a subsequent VL >1000 copies/mL. We also found that among children with VS <50 copies/mL, almost 20% had a later VL 50 to 1000 copies/mL and close to 17% had a VL >1000 copies/mL. Children <12 months were more likely to experience VL rebound; 35% of children <12 months had a VL >1000 copies/mL after VS <50 copies/mL, compared to roughly 16% of children one to five years and 8% of children six to twelve years. In multivariable models, age was the only factor significantly associated with VL rebound after VS <50 copies/mL; children <12 months had an almost twofold increased risk of VL rebound compared to children six to twelve years of age. A study of children starting ART in Kenya also found that infants (median age 3.9 months) had twice the incidence of VL rebound >1000 copies/mL after VS <250 copies/mL compared to older children 17. Sustained VL suppression is critical to achieving optimal treatment outcomes and greater efforts are needed to ensure that all children initiating treatment maintain VS.

There are several important strengths of our analysis, as well as some limitations. This observational cohort enrolled ART‐eligible children at five health facilities in Eastern Cape, South Africa. This setting provides important information about VS among children accessing care at publically supported health facilities who were not part of a clinical trial. While we included three large referral hospitals in the study where sicker children were more likely to seek treatment, we feel that the children included in this analysis are a good representation of those starting ART in this region of South Africa at the time the study was conducted and are also representative of the larger population of children living with HIV initiating treatment in similar settings. The Eastern Cape is an underrepresented region of South Africa with regard to research studies despite having 30% antenatal HIV prevalence thus making this report of outcomes among HIV‐infected children unique 30. In addition, our sample included children ranging from newborns to 12 years of age that allowed us to examine and compare outcomes across age groups. It is also important to note that South Africa was one of the first countries in sub‐Saharan Africa to adopt LPV/r as part of the first‐line regimen for children under three years of age making our data novel with regard to VS outcomes among children receiving the recommended highly potent ARV regimens. The most significant limitation of the analysis is that the study did not conduct VL testing but rather relied on routine care and medical record data. Children may not have received VL measures or may not have had results recorded in a timely fashion which could contribute to the appearance of longer time to VS. Although most children had a VL test at six and twelve months (79% and 85% respectively) after treatment initiation, we used window periods that may have contributed to somewhat imprecise estimates.

## 5 Conclusions

This study of children initiating ART in routine care settings in South Africa highlights the ongoing challenge of late enrolment in HIV care even in a country that has achieved high coverage of PMTCT and EID. We found suboptimal VS rates, with only 64% of children achieving VS <50 copies/mL. In addition, 20% of children who achieved VS experienced VL rebound following suppression and younger children had twice the risk of viral rebound compared to older children. These findings point to important gaps that must be addressed in order to ensure that all children with HIV are identified and that caregivers and children are supported to achieve optimal treatment outcomes.

## Competing interests

None of the authors have competing interests to declare.

## Authors' contributions

CAT, EJA, SA, MM and ER designed the study; CAT, EJA, NS, AM, SA, MN, LP, MM and ER conducted study implementation and/or study oversight; CAT, EJA, KY and CW developed the analytic approach and conducted the analyses; all authors contributed to manuscript writing and review.
